# The Fashion Industry Needs Microbiology: Opportunities and Challenges

**DOI:** 10.1128/msphere.00681-22

**Published:** 2023-02-06

**Authors:** Cristiano Pedroso-Roussado

**Affiliations:** a UNIDCOM/IADE, Lisbon, Portugal; University of Michigan

**Keywords:** microbiology, fashion, synthetic biology

## Abstract

The fashion industry is the second most polluting industry in the world, representing a 2 trillion dollars and growing valuation. Fashion design practices have been perpetuating an industrial-focused approach, which relies mostly in the economic improvement through fast cycles of product development. Additionally, the fashion industry has also been closed to either multidisciplinary or transdisciplinary initiatives outside the scope of the artistic disciplines. Therefore, innovative approaches are needed to solve fashion industrial challenges. One of the most promising fields to tackle current environmental and technological problems in the fashion industry is microbiology. Through the emergent field of synthetic biology, the number of tools and approaches available is increasing and they can already be seen in niche applications. Despite the current advances and urgent need for change, there is still a long way until a more sustainable fashion industry is achieved.

## PERSPECTIVE

Modern fashion practices led to faster production and consumption cycles, which encompasses a greater waste generation (1, 2). In 2015 alone, around 53 million tons of fiber were manufactured; however, 12% were lost during production processes, and 2% were lost during collection and processing. Around 73% was landfilled or incinerated, and only 12% was recycled (3). Another environmental problem of the fashion industry is the extensive use of water and toxic chemicals during manufacturing. Fast fashion has accelerated the problem by increasing the production cycles. Sustainability is a global trend and every brand and textile manufacturer wants to participate (4). Therefore, these industrial players are interested in introducing more sustainable materials and practices (5). Microbiologists have been researching toward a new approach to harness microbes to this end as well, and this knowledge should be appropriated also by designers and entrepreneurs. Current advances in the biotechnology field pushes forward the comprehension and engineering/fabrication of cellular processes deployed by microorganisms (6). Thus, microbiology has the potential to add a whole new range of knowledge and practices to improve the designers’ arsenal and the industrial intellectual property portfolio.

## FASHION INDUSTRY CHALLENGES

The value chain is composed of several steps that mounts the resources necessary to produce fashion products. It is not uncommon that clothes are used only during the first year after being bought, then the common route is disposal, which represents a problem worth more than 140 million euros in the UK alone (7). Additionally, the entire supply chain is producing residues, either due to rejection or discarding, and it may represent around 15% of total fabric manufactured in factories (8).

One endpoint of the fashion industry’s residues is the soil (9–11). Climate change and human activities, such as the use of hazardous agrochemicals, negligent discharge of industrial wastes, maluse of antibiotics, etc. (12–21) are damaging the ecological cycles and degrading soil regulation, which causes the reduction of diversity and the loss of natural resources that could be otherwise exploited (11).

The fashion industry wastewaters represent an additional risk for the environment and human health. The hazardous risk is related to several impairments like photosynthesis rate reduction (22) and throughout the carcinogenic and toxic properties of many utilized dyes that can persist in the environment and be introduced into the food chain (23–25). Therefore, new protocols and processes need to be developed to eradicate the environmental and potential health problems associated with the use of toxic dyes.

Ultimately, as seen in other industries, microbiology is a field that represents a huge potential to guide mitigation and innovation projects for the fashion industry. There are already several projects under way with this mindset; however, the scope has been somehow limited and mostly driven by a marketing ambition.

## MICROBIAL COLLECTIONS, PRODUCTS, AND PROCESSES

The advancement of novel and upgraded techniques for isolation and characterization of microorganisms has increased their study as a tool for biotechnological research, development, and innovation (18). The Microbial Culture Collections (MCC) represents a resource where microorganisms can be investigated for potentially applications (26). Started one century ago, these collections, under management of the Biological Resource Centres (BRC), aim to collect, preserve, distribute, and to disseminate relevant information of microbial strains (18). The work of the BRCs relate to conservation, quality control, and curation; identification, authentication, and taxonomical classification; data gathering, management, and sharing of microorganism-related information and good practices. Microorganisms, DNA, genomes, plasmids, and viable but not yet culturable microorganisms can be sourced in biological or environmental matrices (27, 28). The main purpose of these microbial culture collections is 2-fold. First, it guides the conservation of natural and built habitats through isolation and conservation of microbial diversity. Second, it facilitates the research and development of such microorganisms by the wider public through generation of biotechnological strategies (28). One of the pitfalls of their work is the focus on reference strains, thus limiting the work on the phenotypical variation of other potentially interesting strains. Microbial collections will need more funding to hire experts in taxonomy science, upgrade their infrastructure to work with modern technologies such as next-generation sequencing, and to increase their communication strategies to a wider public, improving their recognition, specifically in terms of their capability to tackle biodiversity challenges (18).

## USE OF LIVING MICROBES

Besides natural products from microbes found in the wild, there are also other approaches under investigation. One of those approaches is to increase the performance of textiles though incorporation of living microbes that can functionalize the garment. In 2015, Yao and colleagues (29) developed a material that responded to body moisture using the absorbent properties of Bacillus subtilis natto. In the same context, the skin microbiome is being widely studied in a project that has gained media attention under the name of “Dr Armpit” (https://drarmpit.com). Broadhead and colleagues (30) recently studied the impact of living microbes in the future of clothing. In their view, the fashion industry must pay attention to the skin microbiome and improve the care related to the antimicrobial finishing of garments, alongside with the potential of functionalizing garments with living microbes that can diminish malodor and eventual skin infections (30). Although, the main limitation of these products is industrial production, and thus, the ability to engineer microbes to surpass that handicap may represent an opportunity for designers and microbiologists alike (31–34).

## BIOMATERIALS

Cellulose is the most abundant material on the planet and bacteria can produce it with diverse properties, like morphology and structure, making it applicable to different uses (35). Several bacteria are cellulose producers, and the members of the recently reclassified genus *Komagateibacter* are the most well studied. Among the fashion designers exploring bacterial cellulose, Suzanne Lee from BioCouture is producing it as a textile material; however, bacterial cellulose may also be developed as fibers and yarns, therefore expanding its application (36). Bacterial cellulose produced using methods inspired by kombucha fermentation constitutes a biofilm or a mat that can be removed from the liquor, rinsed, dried, and potentially tailored to obtain other features such as impermeability (37). This product can perform as a textile and works with aesthetics and properties similar to animal leather (38). However, research is under way to test techniques of garment construction, such as stitching, bonding, and three-dimensional shaping (39–41), and despite its extensive research, intrinsic limitations may hinder its wide use at scale. Thus, caution must be taken when the potential benefits of bacterial cellulose are addressed, mostly because there is no current industrial capacity to deploy enough material to have a global impact. However, there is an increasing interest in addressing the bacterial cellulose scale-up production challenges (e.g., Polybion, personal communication).

Besides bacterial cellulose, mycelium-based composites have also been studied for potential use as fibers (42). Growing in an aqueous substrate, a complex network of fungal hyphae is formed, and its intertwined network of fibers can be recovered, deactivated, and dried for further use (43). Two of the fungi that can be investigated to mycelium production are Ganoderma lucidum and Pleurotus ostreatus (42). One of the first scientists to research mycelium and its fiber application in clothing was Aniela Hoitink (44). She developed a composite product made by *Schizophyllum mycelium* called MycoTEX by Neffa (45). Other initiatives and projects are Mylo from Bolt Threads, MycoFlez from Ecovative, and Reishi Fine Mycelium from MycoWorks. Recently, these initiatives have been gathering media attention (https://www.forbes.com/sites/timnewcomb/2021/04/22/creating-adidas-mushroom-based-stan-smith-mylo-sneakers/?sh=7c632b527c0d). However, challenges remain for the widescale industrial production that could account for the replacement, for instance, of animal leather in fashion manufacturing. Like what is happening in the recent industrial projects producing bacterial cellulose, projects focusing the production of mycelium are highly competitive, having a degree of secrecy and an approach to secure a fair amount of intellectual property outputs. These industrial strategies may constitute a handicap for fast and wide acceptance of the novel materials and processes, slowing down product development in the short term.

## SYNTHETIC BIOLOGY

Apart from natural biomaterials, engineered living materials constitute another frame of opportunity. Once stated as science fiction, these applications might convert into reality since synthetic biology field could develop standardized frameworks to allow the edition of desired properties and functions within complex cellular matrices (46). The potential of synthetic biology can also be addressed by the manipulation of community communication networks, extensive and precise regulation of gene expression, and engineering of syntrophic interactions (47). The predictive understanding of synthetic biology is expanding the applications in several fields, since the computational models are increasing the efficacy of biotechnological developments (46, 48–50).

Rather than using single microorganisms, scientists are now engineering whole communities for biotechnological purposes (47). The progress in synthetic biology has paved the way for construction of microbial consortia aimed at tailored behaviors like bioproduction of drugs, biofuels, and biomaterials in defined environments. The advantages of working with microbial consortia are the division of labor between the community members, spatial organization, and resilience to a myriad of stressors (47, 51–53). Still, the engineering scope of dynamic communities presents a new range of challenges and successful applications are scarce (54–56). Consequently, synthetic biology represents the next opportunity for the industrial revolution. Since we live in a climate emergency (57), all these features can be used for engineering microorganisms and entire microbial communities to reach sustainable goals. However, industrial applications are still in their infancy and further research and pilot projects are needed before a full industrialization can be achieved (58, 59).

## CONCLUSION

The true revolution is to see biology as a manufacturing discipline, and cells as miniaturized factories with the potential to be deployed anywhere at scale with no extra costs to upgrade (46). This is far from what has been observed so far with bio-based material substitutes for a predominantly linear manufacturing industrial value chain. However, designers must embrace the biological disciplines in general, specifically microbiology and biotechnology interpreted as a manufacturing discipline, and take their processes into consideration.

A wider view of design is mandatory, and microbiology represents an escape route from the current crisis. Currently, there may be enough technologies and innovations to solve fashion industry’s main problems; however, there is a long way until sustainability can be achieved. A fast way though is the recommendation of a more intense applied research making use of MCC to solve current industrial challenges, such as wastewater toxicity resulting from garment manufacturing and treatment. Still, the fashion industry value chain is complex, and it will take global cooperation to reinvent it. Ultimately, fashion and other industries must cope with the natural system and not the other way around. To do so, an improved applied research and development of MCC, the extensive use of biomaterials and biomolecules, and bioremediation solutions are necessary to improve the resilience of the current fashion industry models. However, these approaches might not be sufficient to foster an ethical and sustainable change. The promise of synthetic biology to engineer functional and living materials are routes that both designers, scientists, stakeholders, and governmental entities must embrace to speed up the pace for a paradigm shift in design practice, which will acknowledge an update in design education as well. Therefore, the initial cost of synthetic biology projects represents an opportunity which may be addressed by designers, microbiologists, and entrepreneurs alike, and biotechnology companies should evaluate a higher investment in synthetic biology as a competitive advantage. Since the present main business practice is to legally protect inventions, there might be also interest coming from the private funding sector to sponsor more initiatives. On the other hand, the biomaterials field is reaching a tipping point in a way that the next steps are more related to their production scalability and less with their functionalization. The same can be stated about the use of living materials. To solve these challenges, synthetic biology represents a potential solution, and further applications may be expected in the coming years.
